# Monkeypox virus-associated meningoencephalitis diagnosed by detection of intrathecal antibody production

**DOI:** 10.1186/s12879-024-09000-0

**Published:** 2024-01-16

**Authors:** Hansen Karin, Båtshake Ylva, Söderholm Sandra, Pettke Aleksandra, Björkman Per, Sondén Klara

**Affiliations:** 1 Department of Infectious Diseases, Skåne University Hospital, Malmö, Sweden; 2 Department of Microbiology, Public Health Agency of Sweden, Solna, Sweden; 3 Clinical Infection Medicine, Department of Translational Medicine, Lund University, Lund, Sweden; 4 Department of Medicine, Karolinska Institutet, Solna, Sweden

**Keywords:** Mpox, Encephalitis, PCR, Serology, CNS complication, Monkeypox virus

## Abstract

**Background:**

In the 2022 mpox-outbreak most patients presented with mild symptoms. Central nervous system (CNS) involvement has previously been described as a rare and severe complication of mpox; however, diagnostic findings in cerebrospinal fluid (CSF) analysis and neuroimaging studies have only been reported in one case previously.

**Case presentation:**

We report a previously healthy 37-year-old man with mpox complicated by encephalitis. He first presented with painful skin lesions and genital ulcers; polymerase chain reaction (PCR) from the lesions was positive for mpox. Twelve days later he was admitted with fever and confusion. Neuroimaging and CSF analysis indicated encephalitis. The CSF was PCR-negative for monkeypox virus but intrathecal antibody production was detected. He spontaneously improved over a few days course and recovered fully.

**Conclusions:**

This case of mpox-associated encephalitis shows that CNS involvement in mpox infection may have a relatively mild clinical course, and that detection of intrathecal antibody production can be used to establish the diagnosis if CSF monkeypox virus-PCR is negative.

**Supplementary Information:**

The online version contains supplementary material available at 10.1186/s12879-024-09000-0.

## Background

Since May 2022, nearly 90,000 cases of mpox have been reported within a global outbreak [1], with the major proportion of cases affecting men who have sex with men [2]. Apart from this epidemiological pattern of transmission, cases in the global outbreak frequently show a different clinical presentation from that previously described, with a predominance of anogenital lesions, as well as manifestations such as penile oedema, proctitis, and severe pain from skin and mucosal lesions [3]. The course of mpox is usually relatively benign and self-limiting, although within the current outbreak around 6% of patients have required hospitalization [2]. However, the spectrum of severe disease manifestations represents a current knowledge gap. A recent meta-analysis on neurological and psychiatric presentations of mpox reported seizures, confusion, and encephalitis in < 3% of infected individuals [4], but the studies have been small and only one included cerebrospinal fluid (CSF) results and neuroimaging [5].

## Case presentation

The patient was a 37-year-old previously healthy man with a history of primary syphilis who received regular medication with Emtricitabine/Tenofovir as HIV pre-exposure prophylaxis. He presented to an outpatient department with a one-day history of painful genital skin lesions and oral ulcers and reported a casual sexual contact with a man in Spain nine days earlier. Mpox was suspected, and samples obtained from oral and genital lesions were positive for monkeypox virus by PCR (Supplementary material 1). The patient was afebrile and did not show signs of systemic infection. Symptomatic treatment (topical anaesthetics, paracetamol, and ibuprofen) was prescribed.

Over the next week, the patient experienced gradual improvement. On day 9–10 after symptom onset, however, he developed fever, sore throat, headache, and fatigue. On day 12, mental confusion appeared. The patient was found in a parked car, could not provide details about himself, and was brought to the emergency department by ambulance. In the emergency room, he was febrile (39 ºC) with normal respiratory rate. Blood pressure was 120/80 mmHg and heart rate was 92 beats per minute. The neck was supple. He was fully conscious but appeared confused and responded inadequately to questions about his symptoms and previous activities during the day. Neurological examination revealed normal cranial nerve function and intact strength and sensation, and the Glasgow Coma Scale (GCS) was 14/15. The previously described genital and oral ulcers as well as a few additional lesions on the lower extremities and trunk were noted. Computed tomography of the brain was performed with normal findings. Analysis of CSF showed elevated mononuclear cells (22 × 10^^6^/L) suggestive of aseptic meningitis (Table 1). The patient was admitted, and empirical treatment with intravenous acyclovir was initiated.

**Table 1:** Microbiological investigations during hospital admission and at follow-up

Item tested (units)	Laboratory results				Reference values
	Day 1 of admission	Day 3	Day 4	Follow up visit day 38 after admission	
C-reactive protein (mg/L)	12	44			<5
AST (µkat/L)	4.1	3.0		0.64	0.25–0.75
ALT (µkat/L)	2.1	1.0		0.55	0.15–1.1
Hb (g/L)	157 g/L				134–170
Creatinine (µmol/L)	108	84		80	60–105
CSF- mononuclear cells (10^^6^/L)	22		66		<5
CSF- polynuclear cells (10^^6^/L)	3		< 3		<3
CSF- Protein (g/L)	0.59		0.61		0.8 − 0.45
CSF-glucose (mmol/L)	4.0		4.1		
CSF- lactate (mmol/L)	1.9		2.0		<2.2

On the second day after admission, the patient’s condition worsened with psychomotor deceleration and increasing confusion. He moved all extremities spontaneously and had localized pain but gave no verbal or non-verbal response, and a repeated assessment revealed a GCS of 9/15. No focal neurological deficits were observed.

**Fig. 1:**
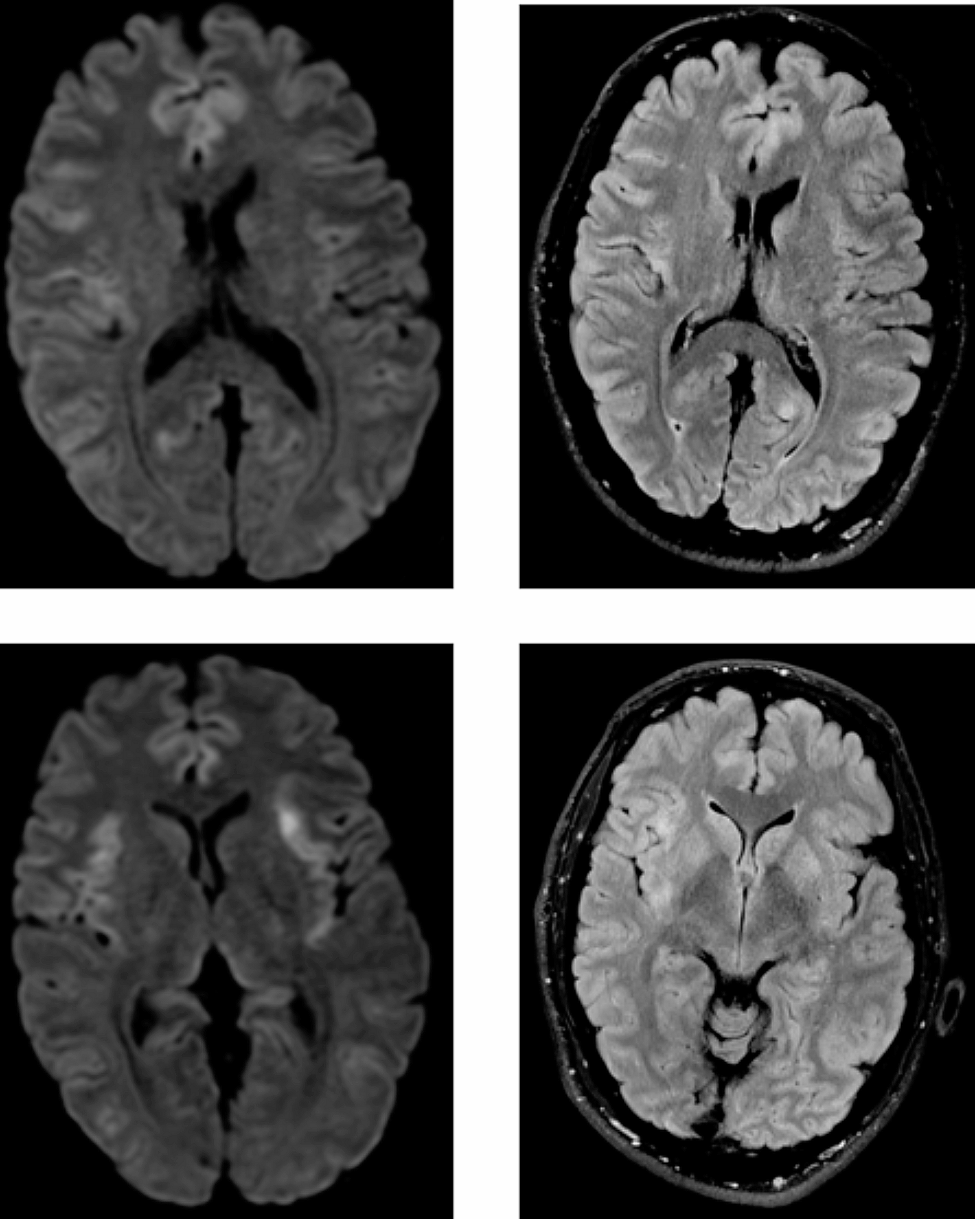
Magnetic resonance imaging (MRI) of the brain. MRI showed a high signal on diffusion weighted imaging in gyrus cinguli, insula and bilateral cortex. Due to agitation, some pictures hade movement artefacts and the MRI were disrupted before contrast injection

Magnetic resonance imaging (MRI) of the brain showed signs of encephalitis with bilateral symmetrical restricted diffusion in the cingulated gyrus and cortical regions of the insula (Fig. 1). An electroencephalogram showed generalized slow waves without epileptiform activity.

The next morning, the patient was awake and responsive, although still showing slight signs of confusion. On day four of admission, he was fully oriented and adequate. A second lumbar puncture was performed. HSV PCR was repeatedly negative, and acyclovir was discontinued. The patient was discharged from hospital five days after admission while reporting mild headache and fatigue.

After discharge, the patient was followed by regular phone calls. He reported complete recovery from day 30, which was confirmed by clinical examination at an outpatient follow-up visit on day 45.

### Microbiological investigations

PCRs specific for monkeypox virus (MPXV) and orthopox virus (OPXV) in the CSF from both sampling time points were negative, as well as PCRs for other neurotropic pathogens (Table 1). Serological analysis was performed by immunofluorescence using MPXV-infected confluent Vero cells. Serum and CSF samples were analysed and serum/CSF ratio was calculated. The presence of EBV antibodies was analysed with the same methodology in order to be able to further differentiate passive transfer of antibodies from signs of intrathecal production of antibodies.

**Fig. 2:**
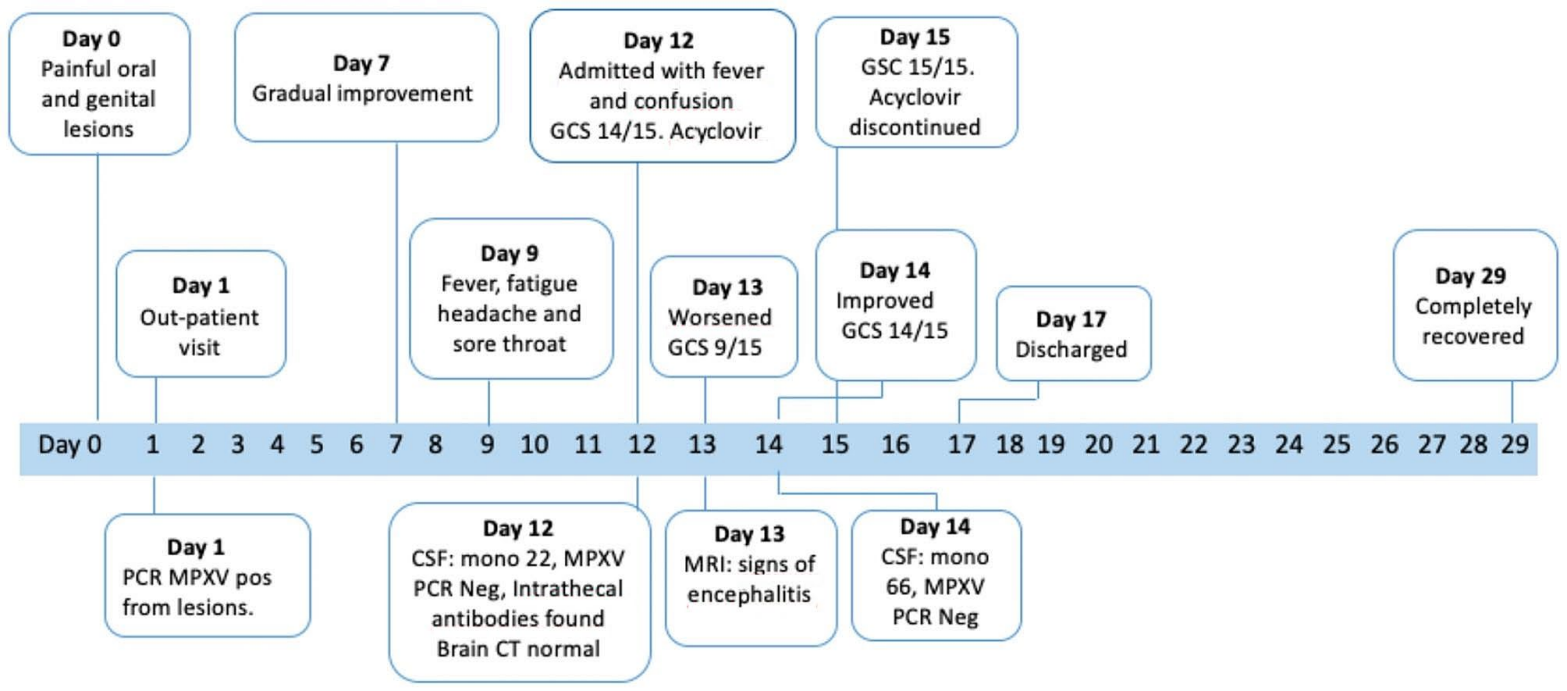
Timeline for the symptoms and investigations performed

Serology for tick-borne encephalitis (TBE) showed a weakly positive IgG signal in serum but with negative IgM, and the CSF showed no signs of production of intrathecal antibodies. *Borrelia burgdorferi* serology was positive for IgG in serum, but both IgG and IgM were negative in the CSF. Tests for autoimmune encephalitis were negative. Further, serology for HIV and hepatis B and C virus were negative. Syphilis serology showed negative VDRL but positive TPPA in both serum and CSF, but with a serum/CSF ratio of 1280 indicating passive antibody transfer rather than local production in the CNS compartment. Serology for HIV was performed 6 and eleven weeks after the hospital admission, with negative results. Microbiological testing is summarized in Table 2, clinical course in Fig. 2.

**Table 2:** Microbiological testing on different days in relation to the day of diagnosis

	Sample type	Day of testing	Pathogen	Analysis	Result
Molecular testing and culture	Vesicle secretion	d1	Monkeypox virus	PCR	Positive
		d12	Herpes simplex virus (HSV) type 1 and 2		Negative
	Throat swab and urine		*C. trachomatis* and *N. gonorrhoeae*		Negative
	CSF	d12	Bacteria	Culture	Negative
			HSV type 1 and 2, varicella zoster virus	PCR	Negative
		d12, d15	Monkeypox virus		Negative
			Orthopox virus		Negative
			*E. coli K1*, *H. influenzae*, *L. monocytogenes*, *N. meningitidis* (capsuled), *S. agalactiae*, *S. pneumoniae*, cytomegalovirus, enterovirus, HSV type 1 and 2, human herpes virus 6, human parechovirus, varicella-zoster-virus and *Cryptococcus neoformans/gattii.*	FilmArray	Negative
Serological testing	CSF	d12	Monkeypox virus	Immunfluorescence (titer IgG)	32 1280
	Serum	d13			
	CSF	d12	EBV	Immunfluorescence (titer IgG)	0
	Serum	d13	EBV		80
	Serum	d13	HIV	Screening (antigen and antibodies)	Negative
				Antigen innotest	Negative
			Hepatitis B	HBsAg	Negative
			Hepatitis C	Anti HCV	Negative
			CMV, EBV	CMV IgG	Positive
				CMV IgM	Negative
				EBV EBNA IgG	Positive
	CSF	d12	*Borrelia Burgdorferi*	IgG	Negative
				IgM	Negative
	Serum	d14		IgG	Negative
				IgM (Screening)	Borderline positive
				IgM (Western Blot)	Negative
	CSF	d12	Autoimmune encephalitis panel	antibodies against NMDA-R, CASPR2, AMPA1/2, LGI1, DPPX, GABARB1/2	Negative
	Serum	d13			
	CSF	d12	Tick-borne encephalitis	Intrathecal antibodies	Negative
	Serum	d15		TBE IgG	Positive
				TBE IgM	Negative
	CSF	d15	Syphilis	TPPA	Positive (serum/CSF quota 1280)*
				VDRL	Negative
	Serum			TPPA	Antibodies positive*
				VDRL	Negative
				Wasserman’s reaction	Negative

* The patient was previously successfully treated for confirmed syphilis infection within the last 6 months, and the serum/CSF ratio was high and interpreted as not consistent with intrathecal antibody production

## Discussion

This case demonstrates an example of the spectrum of symptoms of mpox infection and the diagnostic challenges encountered in patients showing signs of CNS involvement. In our patient, monkeypox virus was not identified by repeated PCR in the CSF. Tests for a broad range of alternative causes of meningoencephalitis were performed with negative results. The patient received acyclovir empirically, but no other antimicrobial or immunomodulatory therapy was administered. Other differential diagnoses were considered; for example, drug-induced aseptic meningoencephalitis. This condition is a diagnosis of exclusion, which has been linked to NSAID use in rare cases [6]. Unfortunately, enterovirus testing was performed only in cerebrospinal fluid and no respiratory tract or fecal samples were collected.

However, serological testing for MPXV antibodies in serum and CSF showed a ratio suggestive of intrathecal antibody production in response to MPXV CNS infection. The findings of antibodies to EBV and syphilis were expected because the patient had been treated for syphilis five months prior to contracting mpox and had serological signs of past EBV infection (presence of EBNA antibodies). Similar patterns of antibody detection paired with negative PCR in CSF occurs in other types of meningoencephalitis caused by known neurotropic pathogens such as TBE [7].

Compared to previously reported cases of MPXV meningoencephalitis, our patient differed in the relatively brief disease course with rapid and prompt resolution of symptoms. To our knowledge, there is only one previous report presenting CSF results, a case report from 2003 of a 6-year-old girl with encephalitis and confirmed MPXV infection from PCR of a skin biopsy who had a negative orthopox PCR for the CSF but positive IgM in the CSF [5]. The same pattern was observed in our patient, with the diagnosis confirmed by signs of intrathecal MPXV antibody production. Our patient did not have any known or suspected immunosuppressive condition, and this may have allowed for early clearance of MPXV in the CNS.

We speculate that CNS involvement within the course of mpox episodes might be more frequent than previously estimated, and that the course of MPXV meningoencephalitis can show a spectrum of manifestations, including mild and self-limiting episodes. In order to elucidate this, lumbar puncture for MPXV diagnostics (PCR, followed by serology in PCR-negative cases) should be considered more broadly in patients with MPXV disease, in particular if CNS symptoms (even mild) occur.

## Conclusion

In summary, we present clinical, microbiological, and radiological data from a previously healthy patient with signs of CNS involvement within an outbreak of mpox infection. Central nervous system complications need to be considered in patients with mpox with compatible clinical manifestations, and serology of CSF can be of diagnostic value.

### Electronic supplementary material

Below is the link to the electronic supplementary material.

**Supplementary Material 1:** Description of DNA extraction and PCR method

## Data Availability

To request data from this study the corresponding author may be contacted.

## Abbreviations

CNS: Central nervous system
CSF: Cerebrospinal fluid
GCS: Glasgow Coma Scale
MPXV: monkeypox virus
MRI: Magnetic resonance imaging
PCR: Polymerase-chain reaction
OPXV: orthopox virus
TBE: tick-borne encephalitis
