# Supplementary material for: Monkeypox virus-associated meningoencephalitis diagnosed by detection of intrathecal antibody production
Source: BMC Infect Dis. 2024 Jan 16;24:94. doi: 10.1186/s12879-024-09000-0 (PMC10792818; doi:10.1186/s12879-024-09000-0)
Supplement: Supplementary file 1 — Supplementary Material 1: Description of DNA extraction and PCR method [file 12879_2024_9000_MOESM1_ESM.docx]

**Supplementary material**

**Description of DNA extraction and PCR method. The PCR has been developed in-house at the Public Health Agency of Sweden**

Total nucleic acid was extracted using QIAamp DNA Blood Mini Kit (QIAGEN, https://www.qiagen.com) largely following manufacturer’s instructions. A 10-minute heating step at 56 degrees was added for virus inactivation before transfer of the sample on the column.

Realtime PCR was performed using TaqMan Fast Advanced Master Mix (Thermo Fisher Scientific, https://www.thermofisher.com). Positive and negative controls as well as inhibition and extraction controls are part of the quality control for each PCR. The method has been developed in-house at the Public Health Agency of Sweden (Solna, Sweden). Primer and probe sequences were as follows:

MPXV_B21R_Probe CCGTAATCCACTTCCT-FAM-MGB

MPXV_B21R Forward GTCTACAGAGTCCAAATCCTCCTCT

MPXV_B21R_ Reverse TGTGGAGGAKA
